# Vaccine Platforms Combining Circumsporozoite Protein and Potent Immune Modulators, rEA or EAT-2, Paradoxically Result in Opposing Immune Responses

**DOI:** 10.1371/journal.pone.0024147

**Published:** 2011-08-30

**Authors:** Nathaniel J. Schuldt, Yasser A. Aldhamen, Daniel M. Appledorn, Sergey S. Seregin, Youssef Kousa, Sarah Godbehere, Andrea Amalfitano

**Affiliations:** 1 Genetics Program, Michigan State University, East Lansing, Michigan, United States of America; 2 Department of Microbiology and Molecular Genetics, Michigan State University, East Lansing, Michigan, United States of America; 3 Department of Biochemistry and Molecular Biology, Michigan State University, East Lansing, Michigan, United States of America; 4 College of Osteopathic Medicine, Michigan State University, East Lansing, Michigan, United States of America; 5 Department of Pediatrics, Michigan State University, East Lansing, Michigan, United States of America; Agency for Science, Technology and Research (A*STAR), Singapore

## Abstract

**Background:**

Malaria greatly impacts the health and wellbeing of over half of the world's population. Promising malaria vaccine candidates have attempted to induce adaptive immune responses to Circumsporozoite (CS) protein. Despite the inclusion of potent adjuvants, these vaccines have limited protective efficacy. Conventional recombinant adenovirus (rAd) based vaccines expressing CS protein can induce CS protein specific immune responses, but these are essentially equivalent to those generated after use of the CS protein subunit based vaccines. In this study we combined the use of rAds expressing CS protein along with rAds expressing novel innate immune response modulating proteins in an attempt to significantly improve the induction of CS protein specific cell mediated immune (CMI) responses.

**Methods and Findings:**

BALB/cJ mice were co-vaccinated with a rAd vectors expressing CS protein simultaneous with a rAd expressing either TLR agonist (rEA) or SLAM receptors adaptor protein (EAT-2). Paradoxically, expression of the TLR agonist uncovered a potent immunosuppressive activity inherent to the combined expression of the CS protein and rEA. Fortunately, use of the rAd vaccine expressing EAT-2 circumvented CS protein's suppressive activity, and generated a fivefold increase in the number of CS protein responsive, IFNγ secreting splenocytes, as well as increased the breadth of T cells responsive to peptides present in the CS protein. These improvements were positively correlated with the induction of a fourfold improvement in CS protein specific CTL functional activity in vivo.

**Conclusion:**

Our results emphasize the need for caution when incorporating CS protein into malaria vaccine platforms expressing or containing other immunostimulatory compounds, as the immunological outcomes may be unanticipated and/or counter-productive. However, expressing the SLAM receptors derived signaling adaptor EAT-2 at the same time of vaccination with CS protein can overcome these concerns, as well as significantly improve the induction of malaria antigen specific adaptive immune responses in vivo.

## Introduction

Malaria is an infectious disease that continues to devastate populations world-wide, causing nearly 1 million deaths annually, and morbidity that overwhelms the medical capabilities of developing countries. Most cases of Malaria are caused by an infection with the protozoan parasite *Plasmodium falciparum (P. falciparum)*. *P. falciparum* infections are responsible for about 80% of all malaria cases and around 90% of all malaria deaths [1]. It is known that prophylactic vaccination of subjects with irradiated sporozoites can result in 94% protection against subsequent malaria infection, a result confirming that induction of protective immunity to malaria antigens can be achieved, if the vaccine utilized is potent enough and expresses the correct antigenic targets [2]. Unfortunately, the large scale production of irradiated sporozoites for this purpose has not been feasible, largely due to the difficulties associated with cGMP production of this class of vaccine.

Significant efforts have been undertaken to create malaria specific subunit vaccines. Some of the most successful malaria vaccine studies to date have attempted to induce adaptive immune responses to the *P. falciparum* CS protein. CS protein is an ∼58 kD surface protein composed of a middle repeat region consisting of multiple NANP repeats that are flanked by a C-terminus containing a thrombospondin-like type I repeat region (TSR) and an N terminal region that assists with liver cell attachment (Figure S1) [3].

CS protein is found on the surface of *P. falciparum* sporozoites and is also expressed in hepatocytes during liver infection by the parasite. Induction of potent cellular immune responses to CS protein by a prophylactic malaria vaccine could potentially eradicate both sporozoites and infected hepatocytes, potentially stopping the infection before clinical symptoms occur.

Multiple studies have demonstrated the importance of CD8^+^ T cell responses in combating murine malaria infections [4], [5], [6], [7]. An oral salmonella vaccine expressing murine malaria derived CS protein was capable of protecting antibody deficient animals [6]. In addition, passive transfer of CD8^+^ T cells that recognize a specific murine malaria CS protein antigen resulted in 100% survival upon sporozoite challenge [5]. Ads expressing murine malaria derived CS protein have been shown to be capable of providing cytotoxic T cell mediated inhibition of parasite liver stage development up to 93% [7]. One of the most successful subunit malaria vaccines to date contain a hepatitis B viral surface protein (HBsAg) CS fusion protein (referred to as RTS,S). Initial formulations of the RTS,S based vaccines demonstrated little protection. Subsequent trials combining several novel “AS” series adjuvants, (the latter consisting of different preparations of monophosphoryl lipid A (MPL) and a plant extract known as QS21), with the HBsAg-CSP fusion protein (referred to as RTS,S/ASO1B) improved induction of CS specific responses, but this level of protection was still limited, suggesting that a more robust CS protein specific immune response may be required to achieve improved protection rates [8], [9], [10], [11], [12], [13], [14].

Recombinant adenoviruses (rAd) can be utilized to induce potent adaptive immune responses to antigens that they are genetically engineered to express. For example, rAd serotypes 5 and 35 expressing CS protein (Ad5.CS and Ad35.CS respectively) are capable of inducing CS protein specific T and B cells in mice similar to the levels induced by the RTS,S/ASO1B adjuvanted vaccine [15], [16]. These levels paralleled results achieved in mice treated with a rAd35 vaccine expressing the *P. Yoelli* derived CS protein, a vaccine that was also found to be capable of providing up to a 92–94% inhibition of liver infection when vaccinated animals were challenged intravenously (IV) with viable *P. yoelli* sporozoites [17]. Combining rAd35CS and rAd5CS CS protein expressing vaccines in a heterologous prime-boost regimen in rhesus monkeys can induce a more potent T and B cell response than use of either vaccine alone [18]. Heterologous prime-boost vaccination regimens utilizing Ad35.CS and RTS,S/ASO1B have also been analyzed and show significant improvement over either vaccine platform alone [19]. However, the utilization of alternative serotype rAds or chimp derived rAds as a vaccine platform may be dangerous, as our studies and those of others have confirmed the increased innate toxicity of non-Ad5 rAds [20]. Therefore, improving the capability of rAd5 vaccines to induce more potent antigen specific adaptive immune responses is a high priority in the drive to find an efficacious malaria vaccine. In this study we sought to improve CS protein specific CMI responses induced by CS protein-expressing rAd5s by co-expression of innate immune response modulating proteins by the vaccine platform.

The innate immune system plays an integral role in augmenting and/or shaping the induction of antigen specific adaptive immune responses [21]. A group of cellular receptors that recognize a variety of pathogen derived antigens, known as the toll-like receptors (TLRs), play a crucial role in identifying pathogen associated molecular patterns (PAMPS), and then augmenting adaptive responses to those PAMPS. We have previously confirmed that rAds ability to induce innate and adaptive responses are dependent upon several TLR's, and that many of these responses are primarily dependent upon MyD88 functionality [22], [23]. We have also recently demonstrated that when rAd5 vaccines engineered to express a novel *Eimeria tenella* derived TLR agonist, rEA, are co-administered with rAd5 vaccines expressing a target antigen there was significant improvement in the ability of the vaccine to induce antigen specific cellular immune responses [24], [25], [26]. Similarly, we have recently confirmed that co-expression of adaptor proteins derived from SLAM receptors signaling pathways can also augment induction of beneficial immune responses to rAd expressed antigens [27]. In the latter instance we utilized the SLAM family of receptors adaptor protein (EAT-2), an adaptor protein known to mediate SLAM receptor signaling in immune cells [27], [28].

In this study, we determined what the impact of modulation of innate immune responses during CS protein presentation would have upon induction of subsequent CS protein specific immune responses *in vivo*. Unexpectedly, use of a TLR agonist uncovered a potent immunosuppressive activity inherent to the combined use of rEA and CS protein, an activity that mitigated induction of any CS protein specific adaptive immune responses. Fortunately, expression of the SLAM receptors adaptor protein EAT-2 overcame and enlightened possible mechanisms underlying the paradoxical CS protein immunosuppressive activity we uncovered when stimulating TLR pathways.

## Methods

### Vector construction

The Open Reading Frame (ORF) of the *P. falciparum* CS protein gene, composed of a codon optimized consensus of several *P. falciparum* CS protein sequences (Figure S1), was incorporated into plasmid pGA4 (GENEART, Burlingame, CA) and excised from pGA4 using endonuclease *NheI* (NEB, Ipswich, MA). The excised portion was subcloned into the pAd Shuttle vector containing a CMV expression cassette. The resulting pAd-CSP shuttle plasmid was linearized with *PmeI* restriction enzyme and homologously recombined with the pAdEasyI Ad5 vector genome as previously described yielding pAd-CSP [29]. Virus was amplified in HEK293 cells. Ad-CSP virus was purified using a CsCl_2_ gradient as previously described [30]. Direct sequencing and restriction enzyme mapping were carried out to confirm the fidelity of the CS protein sequence. Construction of Ad-GFP, Ad-GFP/rEA, and Ad-EAT2 was performed as previously described [26], [27], [31].

### Animal procedures

All animal procedures were approved by the Michigan State University Institutional Animal Care and Use Committee (IACUC). Intravenous injections were performed in 8week old BALB/cJ mice (Jackson Laboratory, Bar Harbor, ME) via retro-orbital sinus in a volume of 200 µL of PBS. 8 weeks old male BALB/cJ mice were injected intramuscularly (IM) into the tibialis anterior of the right hindlimb. Total injected volume was 20 µl. Splenocytes and plasma were collected. All procedures with rAds were performed under BSL-2, and all vector treated animals were maintained in ABSL-2 conditions. Care for mice was provided in accordance with PHS and AAALAC standards.

### Cytokine and chemokine analysis

Plasma levels of cytokines/chemokines were measured by a mouse multiplex kit per manufacturer's instructions (Bio-Rad, Hercules, CA) via Luminex 100 technology (Luminex, Austin, TX) as previously described [22].

### ELISA

ELISA-based antibody assays were completed as previously described [31]. High-binding flat bottom 96-well plates were coated with 0.2 µg of purified CS protein per well in a volume of 100 µL and incubated overnight at 4°C. Plates were washed with PBS-Tween (0.05%) then blocked with blocking buffer (3% bovine serum albumin) for 1 hour at room temperature. Plasma was diluted (1∶50, 1∶100, 1∶200, 1∶400) in blocking buffer.and added to the wells and incubated for 1 hour at room temperature. Wells were washed with PBS-Tween (0.05%) and HRP antibody (Bio-Rad) was added at 1∶4000 dilution in PBS-Tween. Tetramethylbenzidine (TMB) (Sigma-Aldich) was added to each well and the reaction was stopped with 1N phosphoric acid. Plates are read at 450 nm in a microplate spectrophotometer. Subisotyping tittering was completed with a hybridoma subisotyping kit (Calbiochem, La Jolla, CA) with plasma dilutions of 1∶50, 1∶100. 1∶200. 1∶400. Statistical analyses were performed using Student *t*-test.

### Isolation of lymphocytes

Splenocytes from individual mice were prepared by physical disruption of the spleen. The spleen was passed through a sterile 40 µm nylon mesh cell strainer (Fisher Scientific, Pittsburgh, PA). Red blood cells were lysed using ACK lysis buffer (Invitrogen, Carlsbad, CA) remaining cells were resuspended in RPMI 1640 supplemented with 10% FBS and penicillin/streptomycin/fungizone [26].

### ELISPOT analysis

ELISpots were performed in accordance to manufacturer's protocol using the Ready-set Go IFNγ mouse ELISpot kit produced by eBiosciences (San Diego, CA). Splenocytes were stimulated *ex vivo* with 4 µg/mL of the >98% pure CS protein immunodominant peptide NYDNAGTNL (amino acids 43–51 of the CS protein sequence) (GenScript Piscataway, NJ) [32]. A library of 15mers overlapping by 5 amino acids spanning the entire CS protein non-repeating region was constructed and also used to stimulate splenocytes *ex vivo* (Biosynthesis Inc., Lewisville, TX). Spots were counted and photographed by an automated ELISPOT reader system (Cellular Technology, Cleveland, OH). Ready-set Go IFNγ and IL-2 mouse ELISPOT kits purchased from eBioscience (San Diego, CA).

### Cell staining and flow cytometry

Splenocytes were stained with various combinations of the following antibodies: PE-CD69, (3 µg/ml), FITC-CD8a, APC-CD3, APC-Cy7-CD3, Alexa Floure700-CD8a, PerCpCy5.5-CD19, PE-Cy7-NK1.1, PE-Cy7-TNFα, APC-IFNγ (4 µg/ml) (All obtained from BD Biosciences, San Diego, CA), and PerCpCy5.5-IL-2 (4 µg/ml) (BioLegend, San Diego, CA). Cells were incubated on ice with the appropriate antibodies for 30 minutes, washed, and sorted using an LSR II instrument and analyzed using FlowJo software. For intracellular cytokines staining, cells were surface stained, fixed with 2% formaldehyde (Polysciences, Warrington, PA), permeabilized with 0.2% Saponin (Sigma-Aldrich, St. Louis, MO), and stained for intracellular cytokines. Large cells and debris were excluded in the forward- and side-scatter plot, to minimize background levels of staining caused by nonspecific binding of antibodies; we initially stained the cells with CD16/32 FcR III/II antibody. In addition we included the violet fluorescent reactive dye (ViViD, Invitorgen) as a viability marker to exclude dead cells from the analysis [33]. Blood was isolated by retro-orbital bleeds and PBMCs were isolated using Lympholyte-Mammal (Cedarlane, Burlington NC).Tetramer staining of PBMCs was completed using a PE conjugated MHC-I (H2d) tetramer folded with the NYDNAGTNL peptide generated at the NIH Tetramer Core Facility.

### In vivo CTL assay

BALB/cJ were co-vaccinated with equivalent doses of Ad-CSP and either Ad-GFP or Ad-EAT2 (totaling 2×10^8^ vps). At 14 days, syngeneic splenocytes were isolated and pulsed with either an irrelevant peptide or peptide specific to the *Plasmodium falciparum* circumsporozoite antigen (NYDNAGTNL) for 1 hour at 37°C. Irrelevant peptide pulsed cells were stained with 1 µM CFSE (CFSE^Low^) while CS protein-peptides pulsed cells were stained with 10 µM CFSE (CFSE^High^). Naïve and immunized mice were injected with equivalent amount of both CFSE^Low^ and CFSE^High^ stained cells via the retro-orbital sinus. After 18 hours splenocytes were harvested and sorted on an LSRII flow cytometer. FlowJo software was used to determine percentages of CFSE stained cells. % Specific killing  = 1-((% CFSE^High^/% CFSE^Low^) _immunized_/(% CFSE^High^/% CFSE^Low^) _non-immunized_).

### Statistical analysis

Statistically significant differences in ELISpot assays were determined using One Way ANOVA with a Student-Newman-Keuls post-hoc test (p value <0.05). For ELISA analysis, a t-test was used to assess significance between treatments. For multiparameter flow cytometry, a One Way ANOVA with a Student-Newman-Keuls post-hoc test was used. For *in vivo* CTL assay, a One Way ANOVA with a Student-Newman-Keuls post-hoc test was used. All graphs in this paper are presented as Mean ± SD with the exception of graphs of ELISA data which use Mean ± SE. GraphPad Prism software was utilized for statistical analysis.

## Results

### CS protein expressed from rAd5 based vaccines can induce CS protein specific B and T cell responses

**Figure 1:**
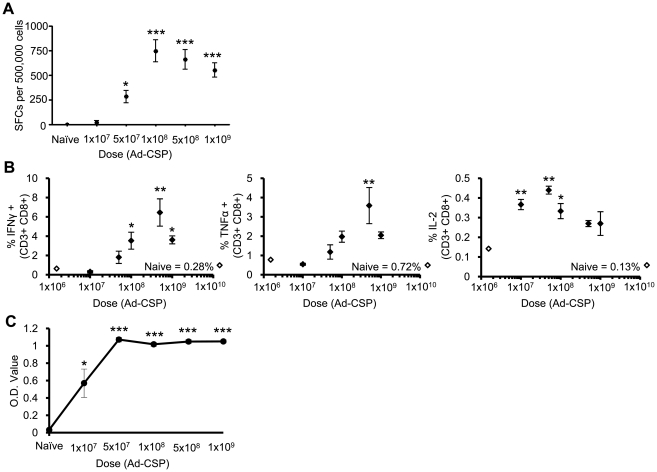
Ad-CSP Stimulates CS protein specific T and B cell responses. CS protein specific immune responses increase in an Ad-CSP dose dependent manner. BALB/cJ mice (n = 3) were injected IM with Ad-CSP ranging from 1×10^7^ to 1×10^9^ vps/mouse, increasing by half logs. 14 days post injection splenocytes and plasma were collected. (A) ELISpot assays were performed to quantify IFNγ secreting cells from splenocytes stimulated with CS protein peptide, NYDNAGTNL, *ex vivo*. (B) IFNγ, TNFα, and IL-2 expression by splenocyte derived CD3^+^ CD8^+^ T cells was analyzed by flow cytometry following *ex vivo* stimulation with NYDNAGTNL. (C) Total IgG against CS protein was assessed by ELISA. The bars represent mean ± SD. Statistical analysis was completed using One Way ANOVA with a Student-Newman-Keuls post-hoc test, *,**,*** denotes significance over naïve, p<0.05, p<0.01, p<0.001.

A rAd5 based vaccine expressing a codon optimized form of the CS protein (Ad-CSP) was constructed (Figure S2), and a dose study was initially performed to assess at what dose optimal CS specific B and T cell responses could be detected. BALB/cJ mice were intra-muscularly (IM) injected with varying doses of Ad-CSP ranging from 1×10^7^ to 1×10^9^ virus particles (vps) per animal. At 14 days post injection (dpi) splenocytes derived from the vaccinated animals were harvested, and exposed to an immunodominant CS protein derived peptide (NYDNAGTNL). Significantly increased numbers of IFNγ secreting splenocytes were noted in Ad-CSP vaccinated mice treated with 5.0×10^7^ to 1.0×10^9^ vps, with peak numbers achieved at a dose of 1.0×10^8^ vps/mouse. Higher Ad-CSP doses resulted in a trend of decreasing, though not significantly less, numbers of spot forming cells (SFCs) (Figure 1A). Interestingly this phenomenon has also been observed by other groups, however an explanation for this phenomenon has yet to be forwarded [15], [34]. These finding were further supported in individual splenocytes derived from the vaccinated animals, where CD8^+^ T cell IFNγ, TNFα, and IL-2 levels were measured by intracellular staining (ICS) using flow cytometry. IFNγ and TNFα production peaked at the 5.0×10^8^ vps/mouse with similar decreasing trend occurring at 1.0×10^9^ vps/mouse. IL-2 producing cells were much lower in percentage, with the greatest numbers being observed as the 5.0×10^7^ vps/mouse dose (Figure 1B). We will further discuss the importance of these findings in the Discussion section.

To determine if Ad-CSP is also capable of stimulating B cell responses specific to the CS protein, plasma was collected from the vaccinated mice and assayed by a IgG CS protein specific ELISA at 14 dpi. Significant increases in CS specific IgG were detected in all mice treated with Ad-CSP with a peak response occurring at 5.0×10^7^ vps/mouse, demonstrating Ad-CSP is capable of stimulating a B cell response against CS protein even at the lowest dose used in the study (Figure 1C).

### The use of a TLR agonist unexpectedly reduces CS protein specific cellular immune responses

**Figure 2:**
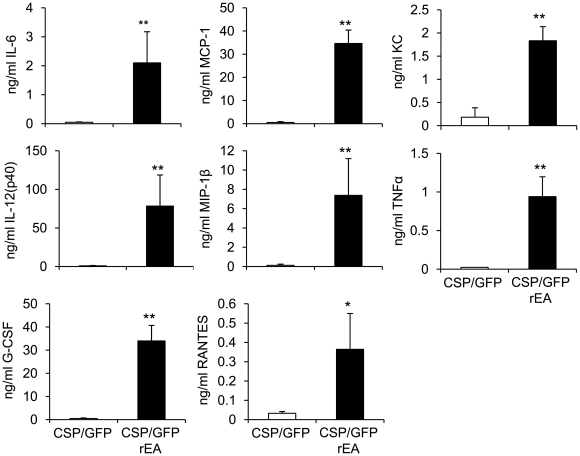
TLR agonist, rEA, induced innate cytokines 6 hours post injection. Co-injection of Ad-GFP/rEA and Ad-CSP stimulated robust expression of innate cytokines and chemokines as compared to the control vaccine. BALB/cJ mice were injected IV with either 3.75×10^10^ vps/mouse of Ad-CSP+Ad-GFP or 3.75×10^10^ vps/mouse Ad-GFPrEA+Ad-CSP. Plasma was collected at 6 hours post injection. Plasma cytokine/chemokine levels were measured with a mouse multiplexed bead array based quantitative system. The bars represent mean ± SD. Statistical analysis was completed using One Way ANOVA with a Student-Newman-Keuls post-hoc test, *,** denotes significance between treatments, p<0.05, p<0.01.

Previous experiments confirm that expressing the TLR agonist rEA from an Ad vector stimulates innate immune responses during Ad mediated vaccination, responses that positively correlated with improved induction of antigen specific adaptive immune responses against several antigens, such as the HIV antigen, Gag [26]. In this study, we sought to utilize rEA to improve induction of CS protein specific immune responses. We first confirmed that expression of rEA along with CS protein facilitated induction of pro-inflammatory innate immune responses, responses we had noted in our previous studies of rEA [26]. Plasma cytokine levels at 6 hours post injection (hpi) in mice co-injected intravenously (IV) with either 3.75×10^10^ vps of Ad-CSP and 3.75×10^10^ vps Ad-GFP/rEA were compared to responses measured after identical co-injections utilizing an Ad-GFP expressing vector (that does not express rEA) as a control (Figure 2). We observed significantly higher levels of IL-6, IL-12(p40), G-CSF, MCP-1, MIP-1β, RANTES, KC, and TNFα in mice treated with Ad-CSP+Ad-GFP/rEA as compared to control virus treated animals, as well as, mock infected animals (Figure 2).

**Figure 3:**
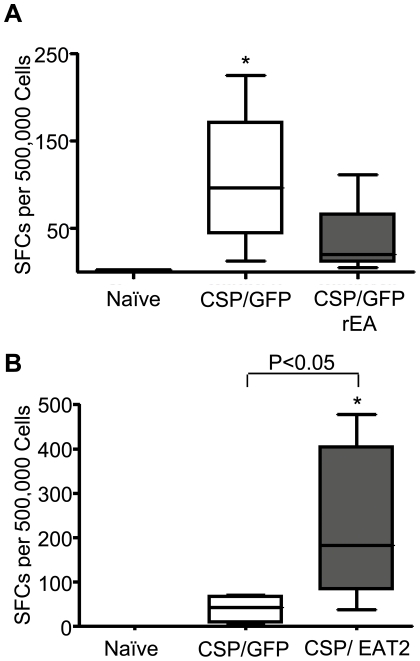
Immuno-modulating proteins conversely affect IFNγ secreting splenocytes. Co-vaccination with Ad-CSP and Ad-EAT2 dramatically increases IFNγ secreting splenocytes in response to stimulation with CS protein epitope, NYDNAGTNL. BALB/cJ mice were injected IM with either 5×10^7^ vps/mouse of Ad-CSP and 5×10^7^ vps/mouse Ad-GFP or 5×10^7^ vps/mouse of Ad-CSP and 5×10^7^ vps/mouse of either (A) Ad-GFP/rEA (n = 5) or (B) Ad-EAT2 (n = 6). Splenocytes were collected 14 days post co-injection. ELISpot were performed on the splenocytes of these mice stimulated with NYDNAGTNL peptide to assess the amount of IFNγ secreting cells. The bars represent mean ± SD. Statistical analysis was completed using One Way ANOVA with a Student-Newman-Keuls post-hoc test,* Denotes significance over naïve animals, p<0.05. Representative figures of two independent experiments.

To assess the impact that these early increases in cytokine and chemokine responses had on cell mediated immune (CMI) responses to CS protein we IM co-injected 5×10^7^ vps/mouse of Ad-CSP and 5×10^7^vps/mouse of Ad-GFP/rEA and compared the induction of CS specific adaptive immune responses to those noted in our control animals receiving 5×10^7^ vps/mouse of Ad-CSP and 5×10^7^ vps/mouse of Ad-GFP IM, or mock infected mice. Splenocytes derived from mock vaccinated animals did not show the presence of CS protein specific CMI responses while Ad-CSP+Ad-GFP confirmed induction of CS protein specific CMI responses using ELISpot analysis (p<0.05) (Figure 3A). However, despite the rEA enhanced activation of the innate immune responses noted in Figure 2, ELISpot analysis of splenocytes derived from Ad-CSP+Ad-GFP/rEA vaccinated animals confirmed a profound lack of induction of average CS protein specific CMI responses, responses that were essentially identical to CS responses measured in naïve mice (p>0.05) (Figure 3A). Previously we have not observed an ablation of CMI responses when CS protein was co-administered with Ads expressing other antigens at these low doses, further suggesting that this effect may be specific to simultaneous TLR stimulation (Figure S3). Despite there being no significant differences between CS protein responses in Ad-CSP+Ad-GFP/rEA treated animals and naïve animals, we did note that in one Ad-CSP+Ad-GFP/rEA animal there was some evidence of an elevated CS protein specific response, independently verifying that this group did in fact receive viable Ad-CSP vector (Figure 3A).

### Augmentation of the innate immune responses via SLAM adaptor EAT-2, improves CS protein specific T-Cell responses

**Figure 4:**
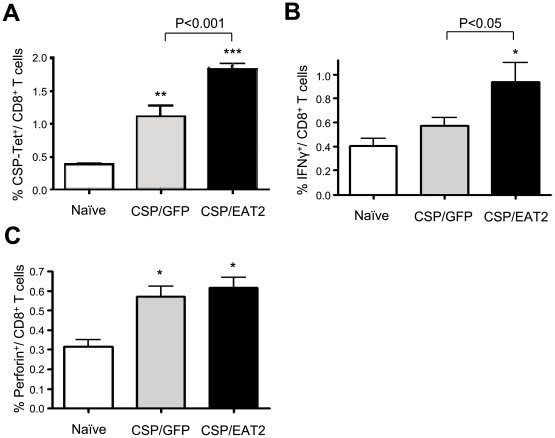
Co-expression of CS protein and EAT-2 stimulates more potent CS protein specific CMI responses. Co-vaccination with Ad-CSP and Ad-EAT2 resulted in increased NYDNAGTNL tetramer positive CD8^+^ T cells as well as improved IFNγ secretion from CD8^+^ T cells. BALB/cJ mice (n = 6) were co-injected IM with 5×10^7^ vps/mouse of Ad-CSP and 5×10^7^ vps/mouse of Ad-EAT2 or 5×10^7^ vps/mouse of Ad-CSP and 5×10^7^ vps/mouse of Ad-GFP. (A) Peripheral Blood Mononuclear Cells (PBMCs) were stained with CD8-Alexa Flour700, CD3-APC-Cy7, and CSP (NYD)-Tetramer. (B–C) Intracellular staining was performed on splenocytes after stimulation with NYDNAGTNL peptide. Cells were stained with CD8-Alexa Flour700, CD3-APC-Cy7, ViViD, IFNg-APC, and Perforin-PE antibodies. The bars represent mean ± SD. Statistical analysis was completed using One Way ANOVA with a Student-Newman-Keuls post-hoc test, *, **, *** denotes significance over naïve animals, p<0. 05, p<0. 01, p<0.001.

Based upon the loss of CS protein responsiveness after utilizing TLR mediated augmentation along with CS protein antigen vaccination we hypothesized that the CS protein may have an ability to mitigate induction of beneficial innate immune responses in the context of excessive, TLR pathway mediated activation as the ablated immune responses were only observed after Ad-GFP/rEA doses exceeded 5×10^6^ vp/mouse (Figure S4). To attempt to test this hypothesis, we made use of a recently described, alternative method for augmenting induction of antigen specific adaptive immune responses, utilizing Ad mediated co-expression of a SLAM receptor signaling pathway adaptor, EAT-2, along with a targeted antigen [27]. To accomplish this we co-injected 5×10^7^ vps/mouse of Ad-CSP and 5×10^7^ vps/mouse of Ad-EAT2, and compared the induction of CS specific adaptive immune responses to those noted in the control mice receiving 5×10^7^ vps/mouse of Ad-CSP and 5×10^7^ vps/mouse of Ad-GFP IM, as well as mock vaccinated mice. Again, splenocytes were collected at 14 dpi and stimulated with the CS derived peptide NYDNAGTNL *ex vivo*. In dramatic contrast to our previous results utilizing the Ad-GFP/rEA and Ad-CSP vaccination strategy, splenocytes from mice co-treated with Ad-CSP and Ad-EAT2 had significantly more IFNγ secreting cells than splenocytes from both mock injected mice as well as mice co-treated with the control vaccine (p<0.05) (Figure 3B). Given these results, we sought to further characterize the EAT-2 dependent improvement in CS specific immune responses by flow cytometry. Peripheral blood mononuclear cells (PBMC) derived from the vaccinated animals were stained with CD3 and CD8 fluorescent antibodies, as well as a NYDNAGTNL peptide loaded tetramer. Ad-CSP+Ad-EAT2 treated mice had significantly higher percentages of CS protein specific tetramer positive CD8^+^cells present in their PBMCs than the percentage noted in the Ad-CSP+Ad-GFP control group (p<0.001) (Figure 4A). CD3^+^ CD8^+^ splenocytes were additionally analyzed for IFNγ and perforin by ICS using flow cytometry. The percent of CD3^+^ CD8^+^ cells that secreted IFNγ was significantly higher in Ad-CSP+Ad-EAT2 treated mice as compared to Ad-CSP+Ad-GFP treated control (p<0.05) (Figure 4B). The percent of CS protein peptide specific CD3^+^ CD8^+^ perforin^+^ cells also tended to be higher in animals given the Ad-EAT2+Ad-CSP vaccination cocktails however this did not reach statistical significance (Figure 4C). To confirm that the differences in the responses observed are not a result of GFP antigens competing with CS protein antigens, but are in fact a direct result of the expression of EAT-2 we injected mice with either Ad-CSP+AdGFP or Ad-CSP + an empty Ad vector (Ad-Null). We observed no differences between the treatments, indicating GFP does not interfere with induction of CS protein specific CMI responses (Figure S5).

**Figure 5:**
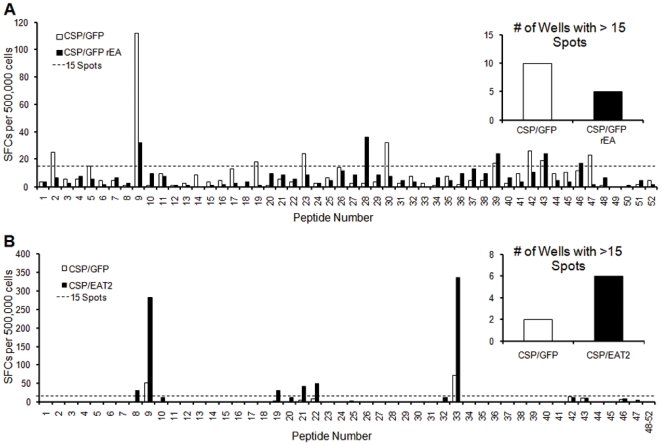
Co-expression of CS protein and EAT-2 increases the breadth of response against CS protein. Increased breadth of response against CS protein epitopes was observed in mice co-vaccinated with Ad-CSP and Ad-EAT2 as compared to the control vaccine. Splenocytes from groups of five BALB/cJ mice were collected and pooled together from 14 days post injection with either innate modulating treatments or control. ELISpots were performed to measure IFNγ secreting cells when stimulated with a CS protein peptide library made up of 52 15mers that overlap by 5 a.a. on either side. Wells that contained more than 15 spots were counted and compared between treatment groups (inset). (A) Mice were co-injected IM with 5×10^7^ vps/mouse of Ad-CSP and 5×10^7^ vps/mouse of Ad-GFP/rEA or 5×10^7^ vps/mouse of Ad-CSP and 5×10^7^ vps/mouse of Ad-GFP. (B) Mice were co-injected IM with 5×10^7^ vps/mouse of Ad-CSP and 5×10^7^ vps/mouse of Ad-EAT2 or 5×10^7^ vps/mouse of Ad-CSP and 5×10^7^ vps/mouse of Ad-GFP. As a negative conrol, naïve splenocytes were also tested against paired peptides from the peptide pool, with an average background of spots per paired peptides being only 2.2 spots.

Increased breadth of CMI responses to a pathogen derived protein has been shown to be beneficial relative to eventual protection against actual pathogen challenge [35], [36], [37]. To detect CMI responses against other peptides present within the CS protein (and therefore to gauge the breadth of response against the whole CS protein) we generated a CS protein specific peptide library. This library consists of 15 mer peptides that overlap each other by 5 amino acids and spans the non-repeating regions of the full length CS protein. At 14 dpi, pooled splenocytes derived from the control or experimental groups of vaccinated animals were stimulated *ex vivo* with one 15mer peptide per well. Mice co-vaccinated with Ad-CSP and Ad-GFP/rEA had an overall lower breadth of response as is evident by the number of wells with more than 15 spots (Figure 5A). In contrast to the response seen in rEA treated animals, animals co-vaccinated with Ad-CSP and Ad-EAT2 demonstrated a dramatic increase in breadth of response to CS derived peptides when similarly analyzed (Figure 5B).

### Co-injection of Ad-CSP and Ad-EAT2 improves the cytolytic activity of CS protein specific T cells in vivo

**Figure 6:**
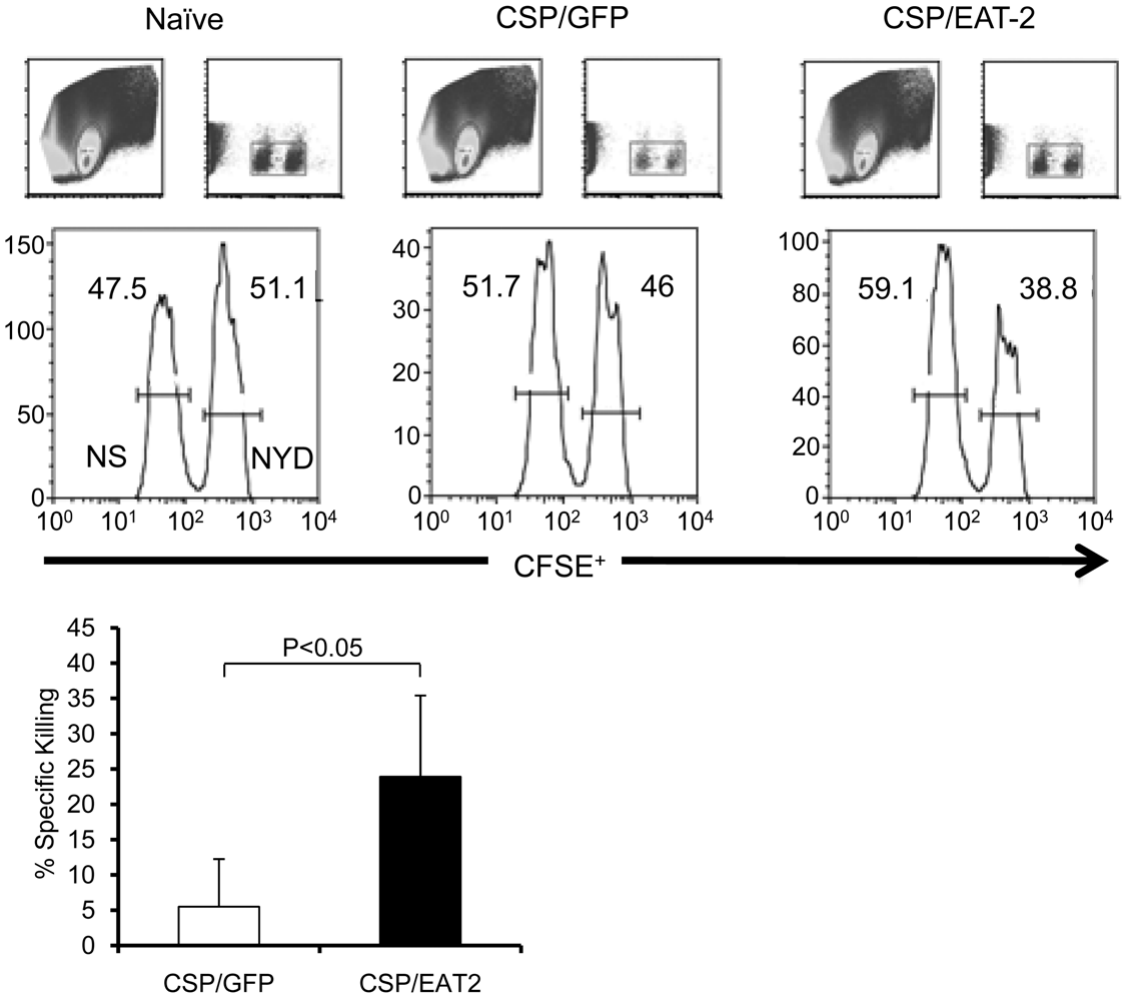
Co-expression of CS protein and EAT-2 increases cytolytic activity of CS protein specific T cells. Co-vaccination of mice with Ad-CSP and Ad-EAT2 increased specific killing cells pulsed with CS protein peptides. BALB/cJ mice (n = 4) were co-injected IM with either 1×10^8^ vps/mouse Ad-CSP and 1×10^8^ vps/mouse Ad-GFP or 1×10^8^ vps/mouse Ad-CSP and 1×10^8^ vps/mouse Ad-EAT2 on Day 0. Day 14 splenocytes were collected from naïve mice and pulsed with either NYDNAGTNL peptide or an irrelevant peptide. NYDNAGTNL pulsed splenocytes were stained with a high concentration of CFSE while splenocytes pulsed with irrelevant peptide were stained with a low concentration of CFSE. Stained splenocytes were then combined in equivalent doses. 8 million cells were then injected IV into naïve, Ad-CSP+Ad-GFP co-vaccinated, or Ad-CSP+Ad-EAT2 co-vaccinated mice. After 18 hrs splenocytes from these mice were collected and analyzed by flow cytometry to assess the amount of NYDNAGTNL specific killing. % Specific killing  =  1-((%CFSE^High^/%CFSE^Low^)_immunized_/(%CFSE^High^/CFSE^Low^)_non-immunized_). * denotes significant difference between treatments p<0.05.

To better assess the functional consequence of the improved CS specific CMI responses noted by expression of EAT-2, we stimulated splenocytes from naïve mice, mice vaccinated with the control vaccine, and mice vaccinated with Ad-CSP+Ad-EAT2 with NYDNAGTNL *ex vivo*, then analyzed them by flow cytometry for CD3^+^, CD8^+^ T cells that were also positive for a degranulation marker, CD107a. Both control treated and Ad-CSP+Ad-EAT2 treated mice demonstrated significantly higher number of CD8^+^, CD107a^+^ T cells than those quantified in naïve mice, indicating increased ability of CD8^+^ T cells to express granules when stimulated with a CS protein epitope (Figure S6). However, the assay was not sensitive enough to measure a difference between the control vaccinated mice and Ad-CSP+Ad-EAT2 vaccinated mice. We then conducted a more sensitive *in vivo* CTL assay [38]. Mice were co-vaccinated with either 1×10^8^ vp/mouse of Ad-CSP and 1×10^8^ vps/mouse of Ad-GFP or 1×10^8^ vp/mouse of Ad-CSP and 1×10^8^ vps/mouse of Ad-EAT2. 14 days later vaccinated mice were treated with CFSE labeled splenocytes that had been incubated with either the NYDNAGTNL peptide, or a non-specific control peptide, and the elimination of NYDNAGTNL pulsed cells (CFSE^high^ cells) was measured by flow cytometry. Based on the calculated percent specific killing, animals vaccinated with Ad-CSP+Ad-EAT2 were more effective at killing cells exposed to the NYDNAGTNL peptide than animals vaccinated with the control Ad-CSP vaccine (Figure 6).

### Induction of CS protein specific antibody responses by Ad-CSP vaccines augmented by rEA or EAT-2 expressing rAds

**Figure 7:**
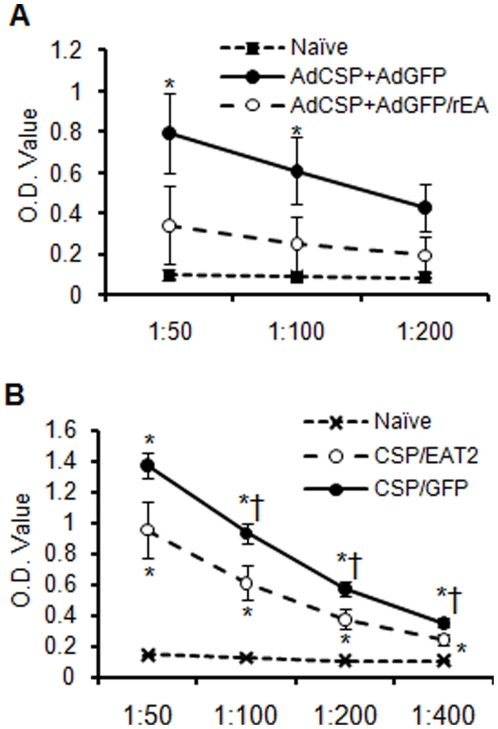
Induction of CS protein specific antibody responses by Ad-CSP vaccines augmented by rEA or EAT-2. Total IgG antibody against CS protein is ablated in Ad-CSP+Ad-GFP/rEA co-vaccinated mice while Ad-CSP+Ad-EAT2 co-vaccinated mice demonstrated significantly more CS protein specific IgG than naïve animals. BALB/cJ mice (n = 5) were co-injected IM with 5×10^7^ vps/mouse of Ad-CSP and 5×10^7^ vps/mouse of Ad-GFP or 5×10^7^ vps/mouse of Ad-CSP and 5×10^7^ vps/mouse of either (A) Ad-GFP/rEA or (B) Ad-EAT2. Plasma was collected at day 14. Total IgG against CS protein in the plasma was measured by ELISA. The bars represent mean ± SD. Statistical analysis was completed using One Way ANOVA with a Student-Newman-Keuls post-hoc test, * Denotes significance over naïve p<0.05. † Denotes significant difference between treatments p<0.05.

CS protein antibody specific ELISAs were also performed on plasma derived from Ad-CSP+Ad-GFP/rEA and Ad-CSP+Ad-GFP treated animals. CS protein specific total IgG antibody levels in control vaccine treated animals were significantly elevated (p<0.05) as compared to naïve animals. However, there was again no significant difference observed in Ad-CSP+Ad-GFP/rEA treated animals when compared to naïve animals (p<0.05) (Figure 7A). Conversely, plasma collected from Ad-CSP+Ad-EAT2 treated animals had significantly higher levels of CS protein specific IgG as compared to levels detected in naïve mice (p<0.05) (Figure 7B). However, the mice receiving the control vaccine treatment had higher total CS protein specific IgG levels than naïve and Ad-CSP+Ad-EAT2 treated animals (p<0.05) (Figure 7B). Further isotyping of IgG was performed, the ratios of T_h_1 to T_h_2 antibody (IgG2a/IgG1) in mice treated with Ad-EAT2+Ad-CSP were similar to the ratio of T_h_1 to T_h_2 antibody in control treated mice in all dilution except 1∶400, indicating that expression of EAT-2 did not induce a T_h_1 or T_h_2 bias in these mice at 14 dpi as measured by this assay (Figure S7). In addition, when measured by ICS, there was no significant difference in the number of likely CD4+ IFNγ expressing T-cells, as the number of CD8^-^ CD3^+^ T cells in Ad-CSP+Ad-EAT2 treated animals and were similar to the numbers of these cells noted in Ad-CSP+Ad-GFP treated animals (Figure S8).

## Discussion

Our earlier works and those of others suggest that activation of the innate immune system can play an important role in beneficially augmenting subsequent antigen specific adaptive immune responses [22], [26], [39], [40], [41]. For example, we previously augmented CMI responses against HIV-Gag by co-injecting a rAd5 vector expressing HIV-Gag with a rAd5 vector expressing a TLR agonist, rEA [26]. Similarly, co-injecting a rAd5 vector expressing HIV-Gag with a rAd5 vector expressing the SLAM receptors adaptor protein EAT-2 also augmented induction of innate immune responses, and improved the induction of HIV-Gag specific T cell responses [27]. As a new approach to increasing the potency of malaria specific vaccines, we now describe the use of adenoviral based vaccines engineered to express malaria derived proteins, simultaneously administered with rAds expressing proteins known to modulate the innate immune system. Most importantly, we have confirmed that rAd mediated expression of a SLAM pathway derived adaptor (EAT-2) can significantly augment the induction of malaria antigen (CS protein) specific CMI responses. This was verified based upon ELISpost analysis of splenocytes (both as to their responsiveness to immunodominant peptides, as well the breadth of these responses to the full length CS protein), ICS staining of cells for IFNγ, and most importantly by a CS protein specific *in vivo* CTL functional assay. EAT-2 expressing vaccines should be considered for use in future malaria vaccine trials attempting to boost malaria antigen specific CMI responses. Furthermore, EAT-2 co-expression allowed for the induction of CS specific antibody responses as well.

In contrast, co-vaccination of mice with a rAd vaccine expressing a TLR agonist simultaneously with a rAd expressing CS protein, actually had the opposite effect, and completely mitigated induction of CS protein specific adaptive humoral and cellular immune responses, as compared to responses typically induced by the rAd vaccines expressing CS protein alone. There could be numerous reasons for these unexpected, paradoxical and potentially disturbing results. A simple reason could be that the increase in pro-inflammatory cytokines caused by rAd mediated expression of the TLR agonist, rEA, could be influencing expression of CS protein from the rAd5 vector. However, this effect would have likely been observed in our previous studies utilizing the same vector combinations, as well as the same TLR or SLAM receptors derived adaptors, but a different target antigen (HIV-Gag). Those studies also confirmed induction of similar innate immune responses to those noted in this study [26], [27]. It is more logical that the CS protein somehow negatively interacts with immune pathways excessively activated by TLR agonists such as rEA, resulting in a complete ablation of CS protein specific CMI responses. This immunosuppressive activity of CS protein appears to only be unveiled after excessive stimulation of TLR pathways, as our use of EAT-2 demonstrated not only avoidance of CS immunosuppressive activity, but also allowed for enhanced induction of CS specific adaptive immune responses.

The CS protein has been specifically confirmed to be capable of outcompeting the transcription factor NF-κB for binding to the nuclear transport protein, importan α, resulting in the downregulation of at least forty NF-κB controlled genes [42]. CS protein was also shown to inhibit NF-κB entry into the nucleus by 75% [42]. As NF-κB is known to control numerous genes involved in pro-inflammatory immune responses, one hypothesis may be that the CS protein can downregulate excessive (TLR-driven) NF-κB transcriptome responses, and result in a dramatically diminished acute inflammatory response, thereby blunting subsequent CS protein antigen specific adaptive immune responses [43]. This may make biological sense, as infection of hepatocytes by malaria sporozoites has been shown to induce the activation of NF-κB in a MyD88 specific manner [44]. Expression of CS protein by the parasite may have evolved to counteract this inflammatory response and prevent excessive induction of malaria specific adaptive immune responses in the infected host. Interestingly, recent studies on an immunosuppressive drug (dehydroxymethylepoxyquinomicin) that specifically interferes with the NF-κB-importan α interaction was shown at lower doses to only modestly affect IL-6 and TNFα levels, while dramatically affecting T_h_1 expansion, results paralleling those noted in our experiments [45].

These notions may also explain our findings, as well results previously reported by others [15], [34]. Those studies and ours verify that at very high doses, rAd vaccines expressing CS protein also show a trend toward diminished induction of CS protein specific CMI responses (Figure 1) [15], [34]. Multiple studies have shown that Ad vectors can also induce NF-κB [46], [47]. Quite possibly, the CS protein immunosuppressive effects are not uncovered until an “NF-κB activation threshold” has been broached, in this instance by use of excessively high doses of rAd vaccines expressing CS protein, or by using more modest doses of the Ad vaccine coupled with potent TLR activation. Further studies will need to be performed to elucidate whether this or other mechanisms may be responsible for our results. Regardless, our data demonstrate the need to consider the impact the inclusion of CS protein derived peptides, or the entire protein along with other immunostimulatory compounds may have upon present and future malaria specific vaccines. Taken together with recent data demonstrating that protection from malaria challenge can be independent of CS protein suggests that the use of CS protein in certain malaria vaccine formulations will have to be carefully considered [48], [49].

In contrast to co-expression of the TLR agonist, co-expression of EAT-2 and CS protein eventuated in the enhanced induction of CMI responses to the CS protein, relative to the use of the Ad-CSP vaccine alone. We have also previously observed a potent CMI response against HIV derived Gag in mice treated with Ad-EAT2+Ad-Gag [27]. Like TLRs, activation of the SLAM receptor pathway in DCs and macrophages can also enhance the production of pro-inflammatory cytokines [50].

The biochemical mechanism and intracellular signaling pathway behind EAT-2's ability to function as a T cell (and possibly a B cell) stimulator in the face of CS protein over expression is not fully elucidated, but is a question that has been unveiled by our studies. SLAM associated proteins like EAT-2 are known to play a role in several novel immunomodulatory pathways, including the SLAM, CD22, and FcγRIIB [51], [52], [53]. These pathways may not be subject to the immune suppressive actions of CS protein possibly by virtue of its specific mode of action relative to NF-κB and/or TLR activation pathways described earlier.

It has been established that greater numbers of CD8^+^ T cells are required to police infected hepatocytes and achieve long term protective immunity against malaria, emphasizing the importance of inducing a large population of CD8^+^ T cells capable of killing [54]. There is some evidence that improved protection is also related to increased breadth of the CMI response in addition to the potency of the CMI response [35], [36], [37]. Here, as an accessory to the increased CMI response, we have demonstrated Ad-EAT2s ability to stimulate increased T cell responses against multiple CS protein epitopes. We not only observed an increase in the percentage of CS protein specific CD8^+^ T cells, but also improved *in vivo* CTL killing of CS pulsed splenocytes from mice treated with Ad-CSP+Ad-EAT2. The use of EAT-2 to augment CSP specific functional CD8^+^ T cells may be of greatest importance in killing *Plasmodium* infected hepatocytes, as these types of responses are not only positively correlated with protective capability, but also may outweigh the need for induction of malaria antigen specific antibody responses [4], [5], [54], [55].

Improvements over sole use of Ad-CSP to induce CS protein antigen specific B cell responses were not achieved in mice treated with either Ad-CSP vaccine cocktail. However, co-vaccination with the Ad-CSP and Ad-EAT2 vectors at least prevented the loss of induction of CS specific antibody responses noted after use of the Ad-GFP/rEA and Ad-CSP vaccine combination. These results did not appear to be due to a skewing from T_h_2 to T_h_1 type antibody response, as measured by IgG1/IgG2a ratios, there were also no observed differences in IFNγ secreting CD8^-^ CD3^+^ T cells between treatment groups. Further research will need to be performed to elucidate the reasons behind the observed antibody responses.

The importance of stimulating a strong cytotoxic T cell response against *P.falciparum* infected hepatocytes is vital in creating a subunit based vaccine that is protective against malaria. With this study we have successfully stimulated a CMI response to CS protein that can overcome CS protein related adaptive immune response ablation and is even more potent than the previous generation of rAd5s expressing CS protein. Incorporation of this new vaccine platform into ongoing or future malaria vaccine trials could potentially achieve the levels of prophylaxis needed to protect vulnerable populations against natural malaria infections. Future studies will need to be performed to assess this platforms ability to protect larger animals challenged with malaria.

## Supporting Information

Figure S1 **CS protein sequence.** The CS protein sequence utilized for constructing the Ad-CSP vaccine was designed based on several known CS protein sequences. The NYDNAGTNL peptide's location is underlined in the sequence. Bold font within the sequence indicates the repeat region of CS protein. The location of the Thrombospondin-like Type 1 repeat region (TSR domain) is indicated by gray font.
(TIF)

Figure S2 **Ad-CSP construction.** Recombinant Ad-CSP was constructed by creating a codon optimized CS protein sequence flanked by NheI sites in a pGA4 plasmid. The sequence was excised with the *Nhe1* and cloned into a pShuttle containing a CMV expression cassette. The resulting plasmid was linearized with *PmeI* and recombined with pAdeasyI Ad5 vector in BJ 5183 cells. pAd-CSP was then purified and linearized with *PacI* enzyme and transfected into HEK 293 cells from which Ad-CSP was purified using cesium gradients.
(TIF)

Figure S3 **CS protein expression does not interfere with antigen specific immune responses against other transgenes at low doses.** Co-vaccination with Ad-gag+Ad-CSP did not result in decreased gag specific immune responses. BALB/cJ mice were injected with 5×10^5^ vp/mouse of Ad-gag and 5×10^7^ vp/mouse of Ad-CSP or 5×10^5^ vp/mouse of Ad-gag and 5×10^7^ vp/mouse of Ad-GFP. Splenocytes were collected 14 dpi and assayed by ELISpot for CS protein peptide (NYDNAGTNL) specific IFNγ secretion (A) or gag peptide (AMQMLKETI) specific IFNγ secretion (B). The bars represent mean ± SD. Statistical analysis for Supplemental Figure 3A included other peptides tested from the peptide library that are not displayed in the graph. Two Way ANOVA with Student-Newman-Keuls post-hoc test (A) or One Way ANOVA with a Student-Newman-Keuls post-hoc test (B) were utilized for statistical analysis. **,*** denotes significance between treatments, p<0.01, p<0.001.
(TIF)

Figure S4 **Ad-GFP/rEA combined with 5×10^7^ vp/mouse of Ad-CSP begins to display a diminished CS protein specific CMI response after a dose of 5×10^6^ vp/mouse.** Only after the dose of Ad-GFP/rEA exceeds 5×10^6^ vp/mouse do we observe a diminished CS specific CMI response when combined with 5×10 vp/mouse of Ad-CSP. BALB/cJ mice were injected with doses ranging from 5×10^6^ to 5×10^8^ vp/mouse of Ad-GFP/rEA combined with 5×10^7^ vp/mouse of Ad-CSP. Splenocytes were collected 14 dpi and were analyzed by flow cytometry for NYDNAGTNL tetramer^+^ CD3^+^ and CD8^+^ cells (A) or ELISpot for CS protein specific IFNγ secretion (B). Statistical analysis was completed using One Way ANOVA with a Student-Newman-Keuls post-hoc test, *** denotes significance between treatments, p<0.01, p<0.001.
(TIF)

Figure S5 **Expression of GFP does not interfere with CS protein specific CMI responses.** Co-injection of Ad-GFP does not interfere with Ad-CSP initiated CS protein specific CMI responses. BALB/cJ mice were co-injected with 5×10^7^ vp/mouse of Ad-GFP and 5×10^7^ vp/mouse of Ad-CSP or 5×10^7^ vp/mouse of Ad-Null and 5×10^7^ vp/mouse of Ad-CSP. Splenocytes were collected 14 dpi and cells were measured for NYDNAGTNL tet^+^, CD3^+^, CD8^+^ T-cells. Both treatments had a higher percentage of CS protein specific tet^+^, CD3^+^, CD8^+^ T-cells than Naïve with no difference observed between Ad-CSP+Ad-Null and Ad-CSP+Ad-GFP. Statistical analysis was completed using One Way ANOVA with a Student-Newman-Keuls post-hoc test, *** denotes significance between treatments, p<0.01, p<0.001.
(TIF)

Figure S6 **Improved degranulation of CD8+ T cells in mice co-vaccinated with Ad-CSP and Ad-EAT2.** Degranulation marker, CD107a, expression in CD8^+^ T cell from mice co-vaccinated with Ad-CSP+Ad-EAT2 or Ad-CSP+Ad-GFP. Splenocytes were collected from BALB/cJ mice 14 days post co-injection of either 5×10^7^ vps of Ad-CSP and 5×10^7^ vps of Ad-GFP or 5×10^7^ vps of Ad-CSP and 5×10^7^ vps of Ad-EAT2. 2×10^6^ splenocytes from naive or mice co-vaccinated with either treatment were stimulated with 2ug NYD-peptide at 37°C for 3 days. Cells were then washed with FACS buffer and stained with CD8-Alexa700, CD107-FITC antibodies and viability dye (ViViD) and ran on LSR-II. % of live CD107+ CD3+ T cells is shown. The bars represent mean ± SD. Statistical analysis was completed using One Way ANOVA with a Student-Newman-Keuls post-hoc test,* Indicates significance over naïve p<0.05.
(TIF)

Figure S7 **Sub-isotype analysis of IgG antibody from plasma of mice co-vaccinated with Ad-CSP and Ad-EAT2.** BALB/cJ mice (n = 6) were co-injected i.m. with 5×10^7^ vps of Ad-CSP and 5×10^7^ vps of Ad-GFP or 5×10^7^ vps of Ad-CSP and 5×10^7^ vps of Ad-EAT2. Plasma was collected at day 14. (A) The amount of CS protein specific IgG subisotypes was measured by ELISA. (B) The ratio of IgG2a/IgG1 was calculated to indicate the T_h_1 to T_h_2 response ratio. The bars represent mean ± SD. Statistical analysis for sub-isotyping (A) was completed using One Way ANOVA with a Student-Newman-Keuls post-hoc test and standard t-test was performed for T_h_1 to T_h_2 ratios (B),* indicates significance over naïve p<0.05. † Indicates significance between treatments p<0.05.
(TIF)

Figure S8 **CD3^+^ CD8^−^ IFNγ^+^ cells respond similarly to both vaccine regimens.** Co-vaccination with Ad-CSP and Ad-EAT2 resulted in similar IFNγ secretion from CD3^+^ CD8^−^ T cells. BALB/cJ mice (n = 6) were co-injected IM with 5×10^7^ vps/mouse of Ad-CSP and 5×10^7^ vps/mouse of Ad-EAT2 or 5×10^7^ vps/mouse of Ad-CSP and 5×10^7^ vps/mouse of Ad-GFP. Splenocytes were stimulation with NYDNAGTNL peptide. Cells were stained with CD8-Alexa Flour700, CD3-APC-Cy7, ViViD, and IFNg-APC. The bars represent mean ± SD. Statistical analysis was completed using One Way ANOVA with a Student-Newman-Keuls post-hoc test.
(TIF)
