# Supplementary figures and images for: Vaccine Platforms Combining Circumsporozoite Protein and Potent Immune Modulators, rEA or EAT-2, Paradoxically Result in Opposing Immune Responses
Source: PLoS One. 2011 Aug 30;6(8):e24147. doi: 10.1371/journal.pone.0024147 (PMC3166157; doi:10.1371/journal.pone.0024147)

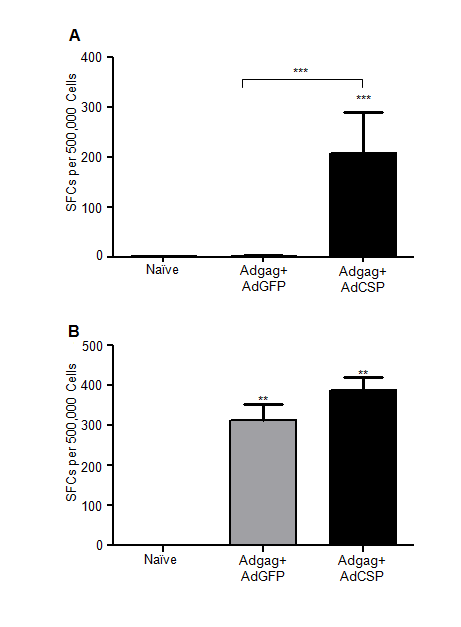

Supplement: Figure S3 — CS protein expression does not interfere with antigen specific immune responses against other transgenes at low doses. Co-vaccination with Ad-gag+Ad-CSP did not result in decreased gag specific immune responses. BALB/cJ mice were injected with 5×10^5^ vp/mouse of Ad-gag and 5×10^7^ vp/mouse of Ad-CSP or 5×10^5^ vp/mouse of Ad-gag and 5×10^7^ vp/mouse of Ad-GFP. Splenocytes were collected 14 dpi and assayed by ELISpot for CS protein peptide (NYDNAGTNL) specific IFNγ secretion (A) or gag peptide (AMQMLKETI) specific IFNγ secretion (B). The bars represent mean ± SD. Statistical analysis for Supplemental Figure 3A included other peptides tested from the peptide library that are not displayed in the graph. Two Way ANOVA with Student-Newman-Keuls post-hoc test (A) or One Way ANOVA with a Student-Newman-Keuls post-hoc test (B) were utilized for statistical analysis. **,*** denotes significance between treatments, p<0.01, p<0.001. (TIF) [file pone.0024147.s003.tif]

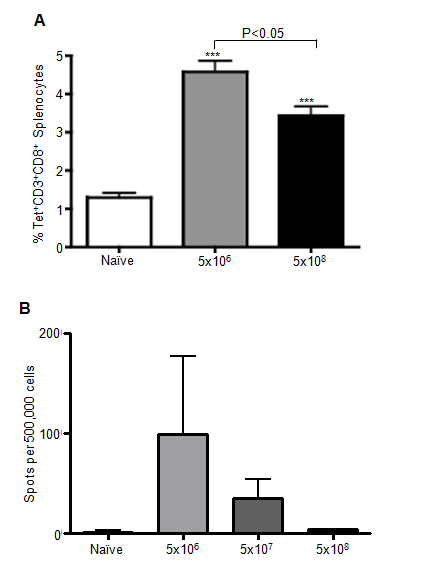

Supplement: Figure S4 — Ad-GFP/rEA combined with 5×10^7^ vp/mouse of Ad-CSP begins to display a diminished CS protein specific CMI response after a dose of 5×10^6^ vp/mouse. Only after the dose of Ad-GFP/rEA exceeds 5×10^6^ vp/mouse do we observe a diminished CS specific CMI response when combined with 5×10 vp/mouse of Ad-CSP. BALB/cJ mice were injected with doses ranging from 5×10^6^ to 5×10^8^ vp/mouse of Ad-GFP/rEA combined with 5×10^7^ vp/mouse of Ad-CSP. Splenocytes were collected 14 dpi and were analyzed by flow cytometry for NYDNAGTNL tetramer+ CD3+ and CD8+ cells (A) or ELISpot for CS protein specific IFNγ secretion (B). Statistical analysis was completed using One Way ANOVA with a Student-Newman-Keuls post-hoc test, *** denotes significance between treatments, p<0.01, p<0.001. (TIF) [file pone.0024147.s004.tif]

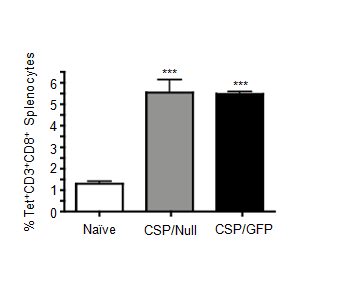

Supplement: Figure S5 — Expression of GFP does not interfere with CS protein specific CMI responses. Co-injection of Ad-GFP does not interfere with Ad-CSP initiated CS protein specific CMI responses. BALB/cJ mice were co-injected with 5×10^7^ vp/mouse of Ad-GFP and 5×10^7^ vp/mouse of Ad-CSP or 5×10^7^ vp/mouse of Ad-Null and 5×10^7^ vp/mouse of Ad-CSP. Splenocytes were collected 14 dpi and cells were measured for NYDNAGTNL tet+, CD3+, CD8+ T-cells. Both treatments had a higher percentage of CS protein specific tet+, CD3+, CD8+ T-cells than Naïve with no difference observed between Ad-CSP+Ad-Null and Ad-CSP+Ad-GFP. Statistical analysis was completed using One Way ANOVA with a Student-Newman-Keuls post-hoc test, *** denotes significance between treatments, p<0.01, p<0.001. (TIF) [file pone.0024147.s005.tif]
